# An enhanced gain non-isolated quadratic boost DC-DC converter with continuous source current

**DOI:** 10.1371/journal.pone.0293097

**Published:** 2023-12-07

**Authors:** Nafis Subhani, Zazilah May, Md Khorshed Alam, Sabrina Mamun

**Affiliations:** 1 Department of Electrical and Electronic Engineering, Leading University, Sylhet, Bangladesh; 2 Department of Electrical and Electronic Engineering, Universiti Teknologi PETRONAS (UTP), Bandar Seri Iskandar, Perak, Malaysia; 3 Department of OS MY Advanced Development & Services, (OS R&D APAC MY ADS), OSRAM Opto Semiconductors (M), Penang, Malaysia; 4 Department of Computer Science and Engineering, International Islamic University Chittagong (IIUC), Kumira, Bangladesh; University of Cagliari

## Abstract

In this paper, a non-isolated quadratic boost DC-DC converter has been proposed. The proposed converter provides high output voltage gain with a lower component count on the structure. In addition, the input side inductor provides continuous source current and the output voltage is positive. Since the proposed topology possesses the continuous source current, it simplifies the filter design at the input side further making the converter suitable for photovoltaic applications. Another important feature of this converter includes the utilization of the same switch ground that omits the additional control power supply in the system design. The detailed mathematical modeling of the proposed topology including the steady state analysis for different modes of operations, voltage stress calculations of the components, and power loss calculations have been precisely demonstrated in this work. The simulation has been carried out in Matlab/Simulink software. Finally, a 250 W experimental prototype has been developed and tested in the laboratory environment and the peak efficiency of the proposed topology has been found 92% at 50% duty cycle, which validates the correctness of the theoretical and simulation outcomes of the proposed work.

## 1 Introduction

**Fig 1:**
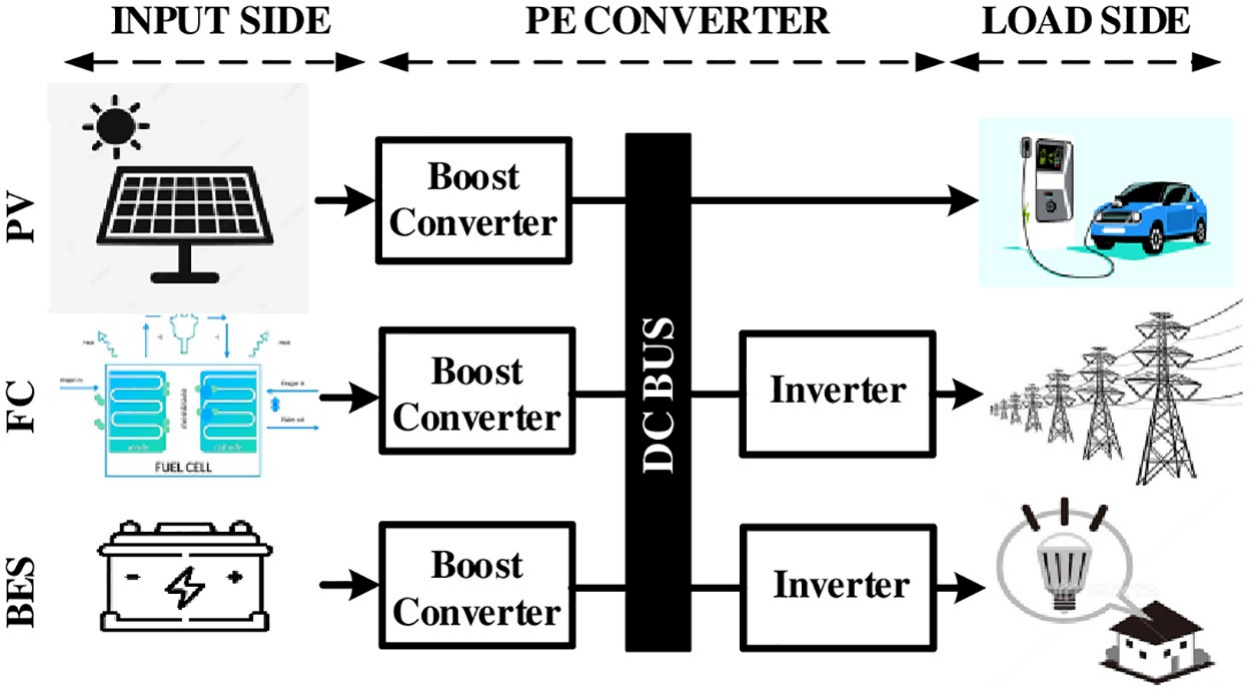
Applications of PE converters.

The gradual depletion of fossil fuels and demand for the green energy to reduce global greenhouse pollution have increased the attention toward the utilization of renewable energy sources (RESs). Since there are unlimited opportunities for green energy, wind, and solar photo-voltaic power generation systems along with other sources (e.g., biomass, tidal, etc.) are contributing extensively to achieve the target for sustainable energy solutions [1, 2]. The technological developments in power electronic converters facilitate opportunities for RESs to be integrated into power grids in grid-connected manners or solely to deliver loads in standalone applications shown in Fig 1. Since output power generated by RESs is intermittent in nature and output voltage needs step-up conversion, power electronic (PE) converters are widely used to meet the grid or load requirement for the smooth coordination of energy generation and distribution [3]. In addition, one of the fast-growing applications is the onboard charger architecture of electric vehicles (EV), where the heart of the system is the DC-DC converters. The recent studies in [4, 5] have focused on the converter’s control and topological development for improving the performance of the charging system. As the demand to establish long-range capabilities with shorter charge times is gradually increasing, the focus on the battery energy storage system (BESS) design is also increasing for the fast EV charging designs. Various EV charging topologies and control studies with BESS can be found in [6, 7]. Primarily, boost and buck-boost converters are utilized to fulfill such requirements but these converters are not capable of attaining desired voltage gain (i.e., high gain) in real-time applications due to their non-ideal operation [8, 9]. To increase the voltage gain of converters, the duty cycle needs to be fixed at a very high percentage which affects the reverse recovery issue of diodes used in these converters while having large stresses (both voltage and current) on switches [10].

The DC-DC boost converters are mainly categorized based on the presence of transformers in the converter structure. A transformer-based structure boosts the voltage by utilizing the suitable turns ratio [11–13] for which the size, weight, and cost of the converter become high because of incorporating bulky transformers. The converter configuration with coupled inductors provides similar facilities while introducing some major issues, e.g., the leakage inductance and switching transients [14–16]. On the other hand, the cascaded converter structure having the boost capability can increase the gain where one converter’s output is fed to the input of the next one [17–19]. Nonetheless, these cascaded structures require to use of a large number of passive components, which further increase the size and cost. Similarly, the interleaved boost converters proposed in [20, 21] have increased the voltage gain with the help of multiple parallel converter structures. The input ripple current has also been improved along with the output power density. However, the switching loss associated with Pulse Width Modulation (PWM) i.e., the ON time control of the semiconductor switches reduces the overall converter efficiency and hence, soft switching solutions have been investigated in [22, 23], where the topologies have significantly improved the converter efficiency at higher switching frequencies. Some other effective solutions to uplift the converter voltage gain are switched inductor/capacitor-based high voltage gain DC-DC converters proposed in [24–28]. Although these converters can generate higher output voltage by the energy transmission between the series-parallel structure of inductors to charge any targeted capacitor, the number of diodes used is usually more in number. As part of continuous improvement, the voltage multiplier cell (VMC) based structures are presented in several literature [17, 29–31] which utilizes switched inductor/capacitor cells to enhance the voltage gain of the converter where converters with switched-capacitors are more popular due to superior voltage gains and smaller physical size.

The quadratic boost converters developed in [28, 31–40], have attained a high gain from the derived structures. Besides, the converters in [36, 38–40] have continuous input current but require a duty ratio of 74% for ensuring the voltage gain of 8. This percentage of the duty cycle causes high electric voltage/current stress on semiconductor devices while reducing the overall efficiency. However, the converter’s gain in [28, 31, 35, 37] has increased more when the duty cycle is nearly 55% to 58% which effectively improves the efficiency of the system. In [36], the converter provides a low voltage gain in the quadratic form with more components on its structure while the feature of the converter in [35] improves the voltage gain along with continuous input current feature but cannot offer the common ground facility on its structure.

The voltage gains are found to be 5 and 6 at 50% duty cycle in [35, 41] respectively. Besides, the topology in [41] gives continuous input current with a common ground feature but 16 components are used in this topology which is very high to attain such a moderate level of voltage gain. Although the converter in [35] ensures the continuous input current, it cannot offer a common ground feature. The topology reported in [42] has the provision for maintaining a common ground and continuous current at the input while producing the same gain as in [35] and utilizing 14 components which are even more than [35].

Furthermore, the cascaded boost converters with quadratic gain nature are proposed in [34, 37], where the number of components is minimized with reduced voltage stress on the passive devices but the voltage gain also remains low in [37] compared to [34]. Also, the semiconductor switches require separate control grounds for both models and ultimately two control power supplies are required. The inclusion of two different power supplies for separate control supplies ground imposes extra ancillary costs for the converter system. By addressing all these aforementioned drawbacks, the main motivation is to keep forth the improvements of the existing models of the cascaded boost converter in the form of quadratic gain. In this paper, an enhanced gain quadratic boost cascaded converter is proposed which has the ability for high voltage conversion while offering a continuous current to its input with a provision of shared switch as well as input to output side ground. The proposed converter exhibits the following key features:

- Improved voltage gain and continuous input current.
- Common switch ground omits separate control supply ground.
- Common ground between the input to output sides.

This paper has the following structure. Section 2 explains the operating principle of the new converter and its performance comparisons with various recent topologies are discussed in Section 3. Section 4 covers the power loss analysis of the proposed converter. Simulation and experimental results of the proposed converter are presented in Section 5. Finally, the paper includes conclusion remarks in Section 6.

## 2 Proposed converter structure

**Fig 2:**
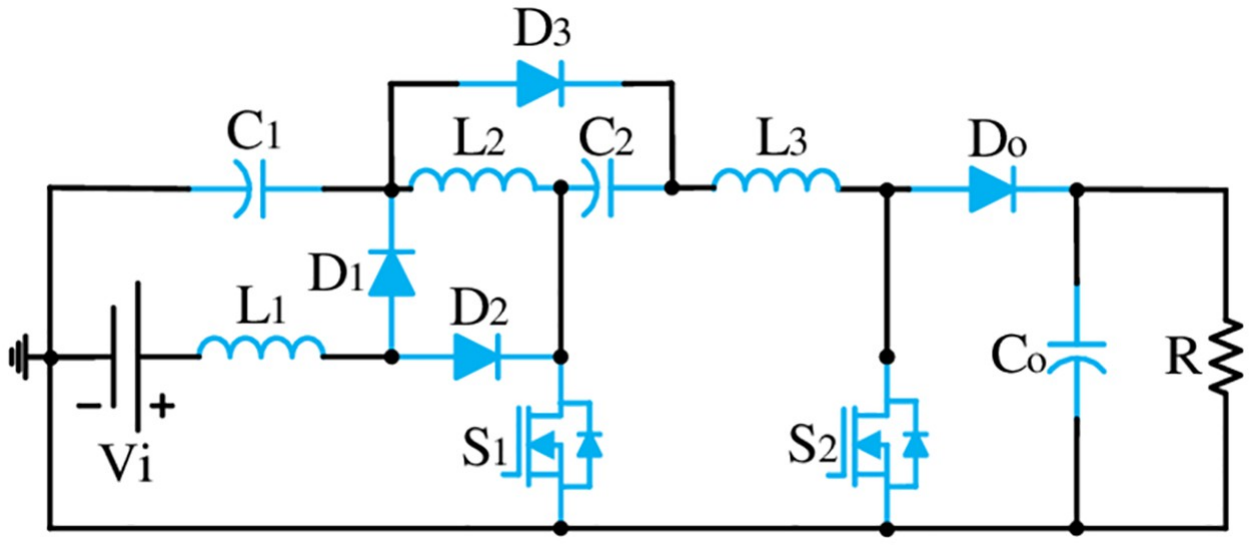
Proposed topology.

**Fig 3:**
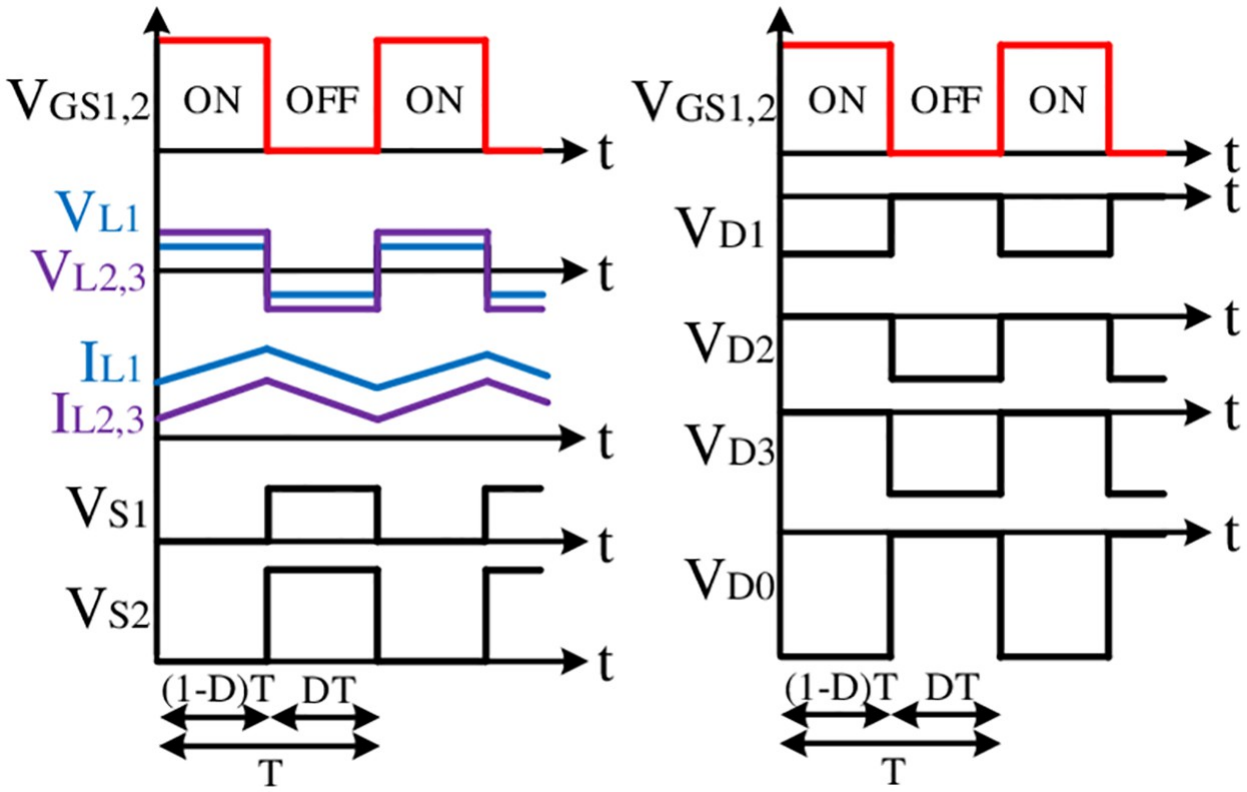
Key switching waveforms.

The proposed converter configuration presented in Fig 2 has only three inductors (*L*_1_, *L*_2_, & *L*_3_), three capacitors (*C*_1_, *C*_2_, & *C*_0_), four diodes (*D*_1_, *D*_2_, *D*_3_, & *D*_0_), and two semiconductor switches (*S*_1_ & *S*_2_). Please note that no voltage doubler circuit accompanying lots of components is used in the proposed topology to uplift its gain. Here, this configuration utilizes only 12 components. In this structure, the first stage of the cascaded boost converter (BC) (*L*_1_ − *D*_1_ − *D*_2_ − *S*_1_ − *C*_1_) has been integrated with the Luo structure (*L*_2_ − *C*_2_ − *D*_3_) followed by the second boost structure (*L*_3_ − *S*_2_ − *D*_0_) which elevates the voltage gain further at a higher level. The conventional cascaded structure utilized a C-D cell in between the first and second cascaded part of the boost converters. Whereas, in the proposed structure, the Luo structure has been integrated after the first BC and the gain has been increased before further integrating with the second BC. The position of the second switch has confirmed the shared ground with the first semiconductor switch that ultimately facilitates a simple control arrangement for the proposed model. Moreover, the position of *L*_1_ with the common connection point of *V*_*i*_, *S*_1_, *S*_2_, *C*_0_, and *R* ensure the continuity in the input current and facilitates the common ground feature in the proposed model. Fig 3 shows the key switching waveform used for this converter where both switches use the same gate pulse and ultimately keep the control simple.

### 2.1 Operating modes

Based on the turn ON and OFF operations of switches, two switching modes are discussed in this subsection.

- **Mode-I**: **ON**

**Fig 4:**
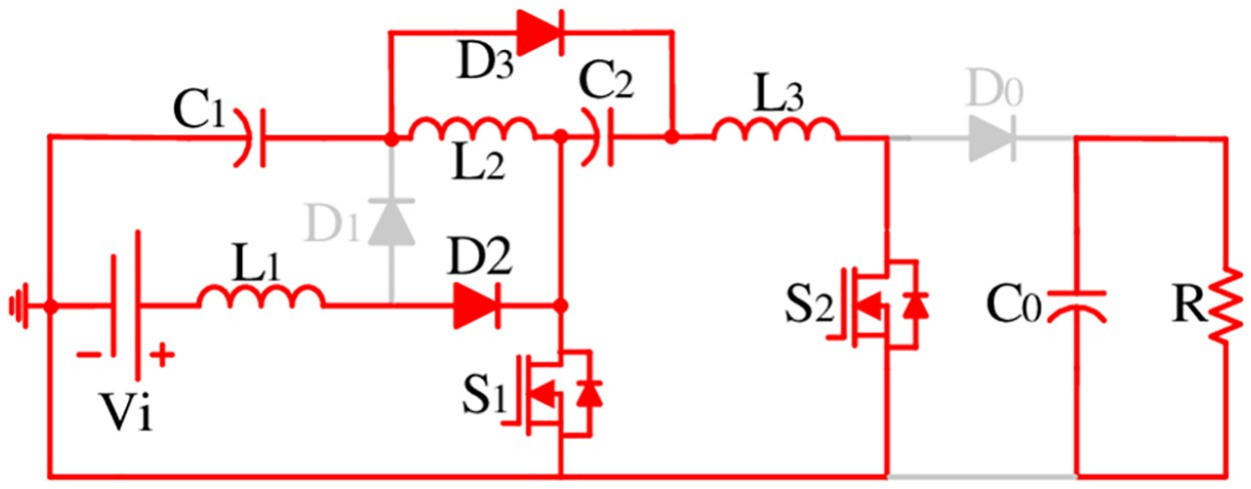
Operating modes: ON.

As demonstrated in Fig 3, the proposed structure operates in Mode-I when the gate pulse is high. In Mode-I, both the switches are ON as can be seen in Fig 4. The diode *D*_2_ and *D*_3_ become forward biased and *D*_1_ and *D*_0_ remain in the reverse biased mode. During this time, all inductors are charged and energy is transferred from capacitors to the inductors and load. By applying Kirchhoff’s voltage law (KVL) during Mode-I, relevant equations can be obtained using Fig 4 as:

$$\begin{array}{c} V_{L1}=L_{1}\frac{dI_{L1}}{dt}=V_{i} \end{array}$$ (1)

$$\begin{array}{c} V_{L2}=L_{2}\frac{dI_{L2}}{dt}=V_{C1} \end{array}$$ (2)

$$\begin{array}{c} V_{L3}=L_{3}\frac{dI_{L3}}{dt}=V_{C2} \end{array}$$ (3)

where *V*_*L*1_, *V*_*L*2_, and *V*_*L*3_ are voltages across respective inductors denoted by subscripts; *I*_*L*1_, *I*_*L*2_, and *I*_*L*3_ are currents through respective inductors denoted by subscripts; *V*_*C*1_ and *V*_*C*2_ voltages across respective capacitors denoted by subscripts; and *V*_*i*_ is the input supply voltage.

- **Mode-II**: **OFF**

**Fig 5:**
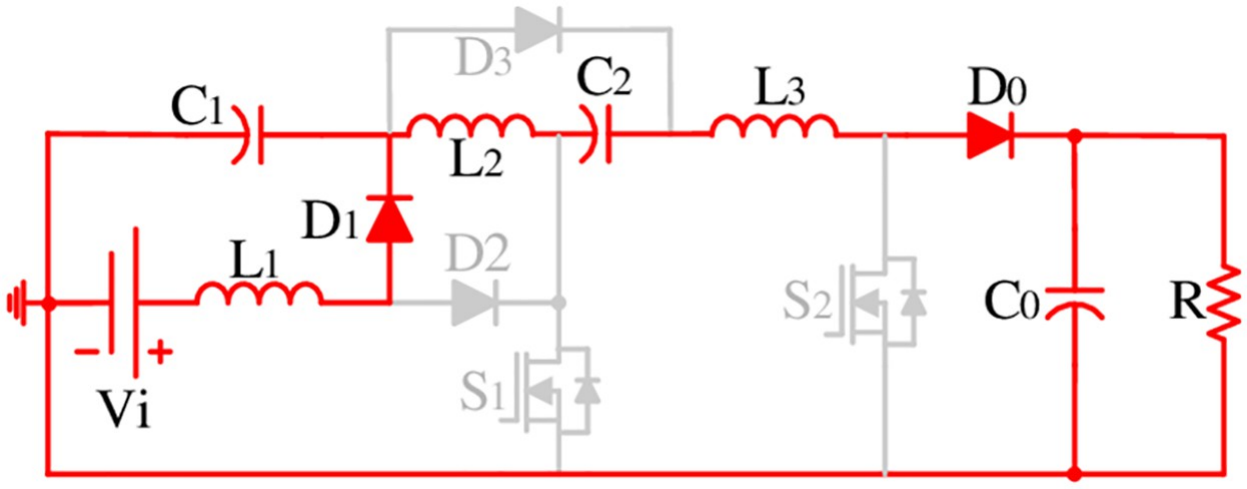
Operating modes: OFF.

Mode-II can be explained from Fig 5 where both switches are OFF and diode *D*_1_ and *D*_0_ become forward biased while both *D*_2_ and *D*_3_ become reverse biased. During this operating mode, all inductors release the stored energy and capacitors start their charging phase. By applying KVL during Mode-II, relevant equations can be obtained using Fig 5 as:

$$\begin{array}{c} V_{L1}=L_{1}\frac{dI_{L1}}{dt}=V_{i}-V_{C1} \end{array}$$ (4)

$$\begin{array}{c} V_{L2}=L_{2}\frac{dI_{L2}}{dt}=\frac{2V_{C1}-V_{0}}{2} \end{array}$$ (5)

$$\begin{array}{c} V_{L3}=L_{3}\frac{dI_{L3}}{dt}=\frac{2V_{C2}-V_{0}}{2} \end{array}$$ (6)

where *V*_0_ is the voltage across *C*_0_ which is also the load or output voltage. Based on these operating modes, the calculation of the voltage gain is presented in the subsection below.

### 2.2 Voltage gain calculation

During the steady state condition, the average voltages across *L*_1_ and *L*_2_ are zero. By applying the voltage-second balance principle on inductors (*L*_1_ and *L*_2_) and using Eqs (1)–(3) and (4)–(6) for two different operating modes, it can be determined that:

$$\begin{array}{c} DV_{i}+\left(1-D\right)\left(V_{i}-V_{C1}\right)=0 \end{array}$$ (7)

$$\begin{array}{c} DV_{C1}+\left(1-D\right)\frac{\left(2V_{C1}-V_{0}\right)}{2}=0 \end{array}$$ (8)

where *D* denotes the duty cycle. Voltages across capacitors (*C*_1_ and *C*_2_) can be obtained using Eqs (1)–(3) to (8) as:

$$\begin{array}{c} V_{C1}=V_{C2}=\frac{1}{\left(1-D\right)}V_{i} \end{array}$$ (9)

Eqs (7) to (9) can be utilized to determine the voltage gain (*M*) as:

$$\begin{array}{c} M=\frac{V_{0}}{V_{i}}=\frac{2}{{\left(1-D\right)}^{2}} \end{array}$$ (10)

Furthermore, the required duty cycle to get the suitable output voltage for an input voltage can be determined as:

$$\begin{array}{c} D=\frac{V_{0}}{V_{i}}=1-\sqrt{\frac{2V_{i}}{V_{0}}} \end{array}$$ (11)

Using this duty cycle, stress (voltage and current) for different components is discussed next.

### 2.3 Stress calculations

**Fig 6:**
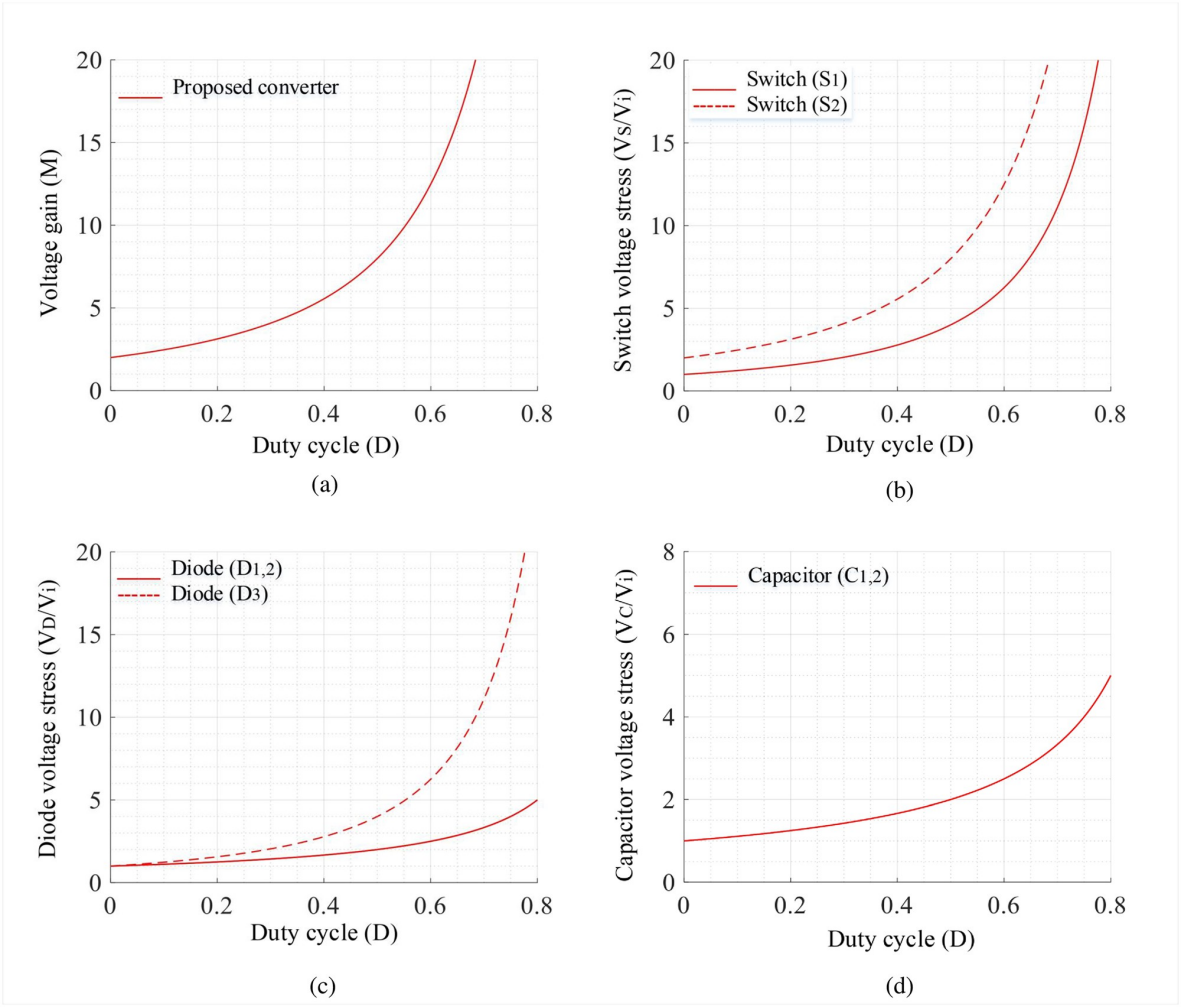
Characteristics curves of proposed converter: (a) *D* vs *M*, (b) *D* vs *V*_*S*1,2_, (c) *D* vs *V*_*D*1,2,3_, and (d) *D* vs *V*_*C*1,2_.

When semiconductor switching elements are OFF in the converter operation, the voltages imposed on diodes (*D*_1_, *D*_2_, *D*_3_, & *D*_0_) and switches (*S*_1_ & *S*_2_) are considered as the voltage stress on these particular components. In contrast, the current that flows during the ON condition of the device is responsible for the current stress of the component. The expressions for voltage stresses on switches and diodes will be as:

$$\begin{array}{c} V_{S1}=\frac{1}{{\left(1-D\right)}^{2}}V_{i} \end{array}$$ (12)

$$\begin{array}{c} V_{S2}=\frac{2}{{\left(1-D\right)}^{2}}V_{i} \end{array}$$ (13)

$$\begin{array}{c} V_{D1}=V_{D2}=\frac{1}{\left(1-D\right)}V_{i} \end{array}$$ (14)

$$\begin{array}{c} V_{D3}=\frac{2}{\left(1-D\right)}V_{i} \end{array}$$ (15)

$$\begin{array}{c} V_{D0}=\frac{2}{{\left(1-D\right)}^{2}}V_{i} \end{array}$$ (16)

where *V*_*S*1_ and *V*_*S*2_ are voltages across *S*_1_ and *S*_2_, respectively; and *V*_*D*1_, *V*_*D*2_, *V*_*D*3_, and *V*_*D*0_ are voltages across *D*_1_, *D*_1_, *D*_1_, and *D*_0_, respectively. The characteristics curves of the proposed converter for the gain and stress are generated using Eqs (9)–(15) and shown in Fig 6. Similarly, the current stresses of switches and diodes will be as:

$$\begin{array}{c} I_{S1}=\frac{4I_{0}}{{\left(1-D\right)}^{2}} \end{array}$$ (17)

$$\begin{array}{c} I_{S2}=\frac{I_{0}}{\left(1-D\right)} \end{array}$$ (18)

$$\begin{array}{c} I_{D1}=I_{D2}=\frac{2I_{0}}{{\left(1-D\right)}^{2}} \end{array}$$ (19)

$$\begin{array}{c} I_{D3}=\frac{2I_{0}}{D(1-D)} \end{array}$$ (20)

$$\begin{array}{c} I_{D0}=I_{0} \end{array}$$ (21)

where *I*_*S*1_ and *I*_*S*2_ are currents flowing through *S*_1_ and *S*_2_, respectively; and *V*_*D*1_, *V*_*D*2_, *V*_*D*3_, and *V*_*D*0_ are currents flowing through *D*_1_, *D*_1_, *D*_1_, and *D*_0_, respectively. Considering the lossless condition, the output power (*P*_0_) will be similar to that of the input power (*P*_*i*_) for which it can be written as:

$$\begin{array}{c} P_{i}=P_{0}=V_{i}I_{i}=V_{0}I_{0}= \end{array}$$ (22)

$$\begin{array}{c} \frac{I_{i}}{I_{0}}=\frac{V_{0}}{V_{i}}=M=\frac{2}{{\left(1-D\right)}^{2}} \end{array}$$ (23)

$$\begin{array}{c} I_{i}=I_{L1}=\frac{2}{{\left(1-D\right)}^{2}}I_{0} \end{array}$$ (24)

$$\begin{array}{c} I_{L2,3}=\frac{1}{\left(1-D\right)}I_{0} \end{array}$$ (25)

where *I*_*i*_ and *I*_0_ are input and output currents, respectively. The switching frequency and duty cycle can be used to determine the design parameters related to different components as discussed next.

### 2.4 Calculations of design parameters

The basic formula to express the voltage equation of the inductor can be expressed as follows:

$$\begin{array}{c} V_{L}=L\frac{d_{iL}}{dt} \end{array}$$ (26)

Considering the ON time (*D*) of inductor (*L*_1_), the ripple current (Δ*I*_*L*1_) and switching frequency (*f*_*S*_), the voltage Eq 26 can be expressed as follows:

$$\begin{array}{c} V_{L1}=L_{1}\frac{d_{iL1}}{dt} \end{array}$$ (27)

$$\begin{array}{c} L_{1}=\frac{V_{L1(ON)}DT_{S}}{\Delta I_{L1}} \end{array}$$ (28)

As the inductor voltage for (*L*_1_) is (*V*_*i*_) during the ON time operation, the equation can be written as,

$$\begin{array}{c} \Delta I_{L1}=\frac{V_{i}D}{L_{1}\;f_{S}} \end{array}$$ (29)

$$\begin{array}{c} L_{1}=\frac{V_{i}D}{\Delta I_{L1}\;f_{S}} \end{array}$$ (30)

According to the aforesaid model, the inductance for inductor (*L*_2_) and (*L*_3_) can be expressed by the following expressions where the ON time voltage of inductors (*L*_2,3_) was (*V*_*C*1_):

$$\begin{array}{c} L_{2,3}=\frac{V_{L2,3(ON)}DT_{S}}{\Delta I_{L2,3}} \end{array}$$ (31)

$$\begin{array}{c} L_{2}=L_{3}=\frac{V_{C1}D}{\Delta I_{L2,3}\;f_{S}} \end{array}$$ (32)

The basic formula to express the charging current equations of the capacitor can be expressed as follows:

$$\begin{array}{c} I_{C}=C\frac{d_{vC}}{dt} \end{array}$$ (33)

Again, for capacitor (*C*_1_), the expression can be reconstructed as follows:

$$\begin{array}{c} I_{C1}=C_{1}\frac{d_{vC1}}{dt} \end{array}$$ (34)

The capacitor (*C*_1_) voltage ripple (Δ*V*_*C*1_) during the OFF time (1 − *D*) operation is used to determine the following equation:

$$\begin{array}{c} \Delta V_{C1}=\frac{I_{C1}\left(1-D\right)T}{C_{1}} \end{array}$$ (35)

As the capacitor current for (*C*_1_) is (*I*_*L*1_ − *I*_*L*2_) during the OFF time, hence the equation can be further expressed as,

$$\begin{array}{c} \Delta V_{C1}=\frac{\left(I_{L1}-I_{L2}\right)\left(1-D\right)}{C_{1}\;f_{S}} \end{array}$$ (36)

By substituting the equations from 24 and 25 to 36, the expression of the capacitance for capacitor (*C*_1_) can be written as follows:

$$\begin{array}{c} \Delta V_{C1}=\frac{\left[\frac{2I_{0}}{{\left(1-D\right)}^{2}}-\frac{I_{0}}{\left(1-D\right)}\right]}{C_{1}\;f_{S}}\left(1-D\right) \end{array}$$ (37)

By substituting the equation from 21 to 37, the further expression of the capacitance for capacitors (*C*_1,2_) can be written as follows:

$$\begin{array}{c} C_{1}=C_{2}=\frac{\left(1+D\right)V_{0}}{\left(1-D\right)\Delta V_{C1}Rf_{S}} \end{array}$$ (38)

where *T*_*S*_ = 1/*f*_*S*_ is the switching period; Δ*I*_*L*1_, Δ*I*_*L*2_, and Δ*I*_*L*3_ are ripple currents for *L*_1_, *L*_2_, and *L*_3_, respectively; and Δ*V*_*C*1_ and Δ*V*_*C*2_ represent ripple voltages for *C*_1_ and *C*_2_, respectively. Based on these equations, the suitable values for inductors and capacitors can be chosen using the desired values of *D*, *f*_*S*_, *V*_*i*_, *V*_0_, and *R*. The boundary conditions for different factors are discussed in the following subsection.

### 2.5 Boundary conditions

The boundary conditions for determining the continuous conduction mode (CCM) of the proposed topology can be determined by setting the currents flowing through the inductors as greater than zero. Hence, the minimum inductance value should be considered for ensuring the operation in the CCM mode. To find the minimum inductor value, the following expressions are used:

$$\begin{array}{c} I_{L1}=\frac{2I_{0}}{{\left(1-D\right)}^{2}} \end{array}$$ (39)

$$\begin{array}{c} \Delta I_{L1}=\frac{V_{i}D}{L_{1}\;f_{S}} \end{array}$$ (40)

$$\begin{array}{c} I_{L1(min)}=I_{L1}-\frac{\Delta I_{L1}}{2} \end{array}$$ (41)

$$\begin{array}{c} I_{L1}\geq \frac{D{\left(1-D\right)}^{4}R}{8f_{S}} \end{array}$$ (42)

$$\begin{array}{c} I_{L2}=\frac{I_{0}}{\left(1-D\right)} \end{array}$$ (43)

$$\begin{array}{c} \Delta I_{L2}=\frac{V_{C1}D}{L_{2}\;f_{S}} \end{array}$$ (44)

$$\begin{array}{c} I_{L2(min)}=I_{L2}-\frac{\Delta I_{L2}}{2} \end{array}$$ (45)

$$\begin{array}{c} I_{L2}\geq \frac{D{\left(1-D\right)}^{2}R}{4f_{S}} \end{array}$$ (46)

Since *I*_*L*2_ = *I*_*L*3_, the calculations are same for *L*_2_ and *L*_3_. The normalized inductor time constant (*τ*) for inductor *L*_1_ will be as:

$$\begin{array}{c} \tau =\frac{8L_{1}\;f_{s}}{R} \end{array}$$ (47)

Using Eqs (39)–(42), the boundary of *τ* (i.e., *τ*_*B*_) will be as:

$$\begin{array}{c} \tau_{B}=D{\left(1-D\right)}^{4} \end{array}$$ (48)

**Fig 7:**
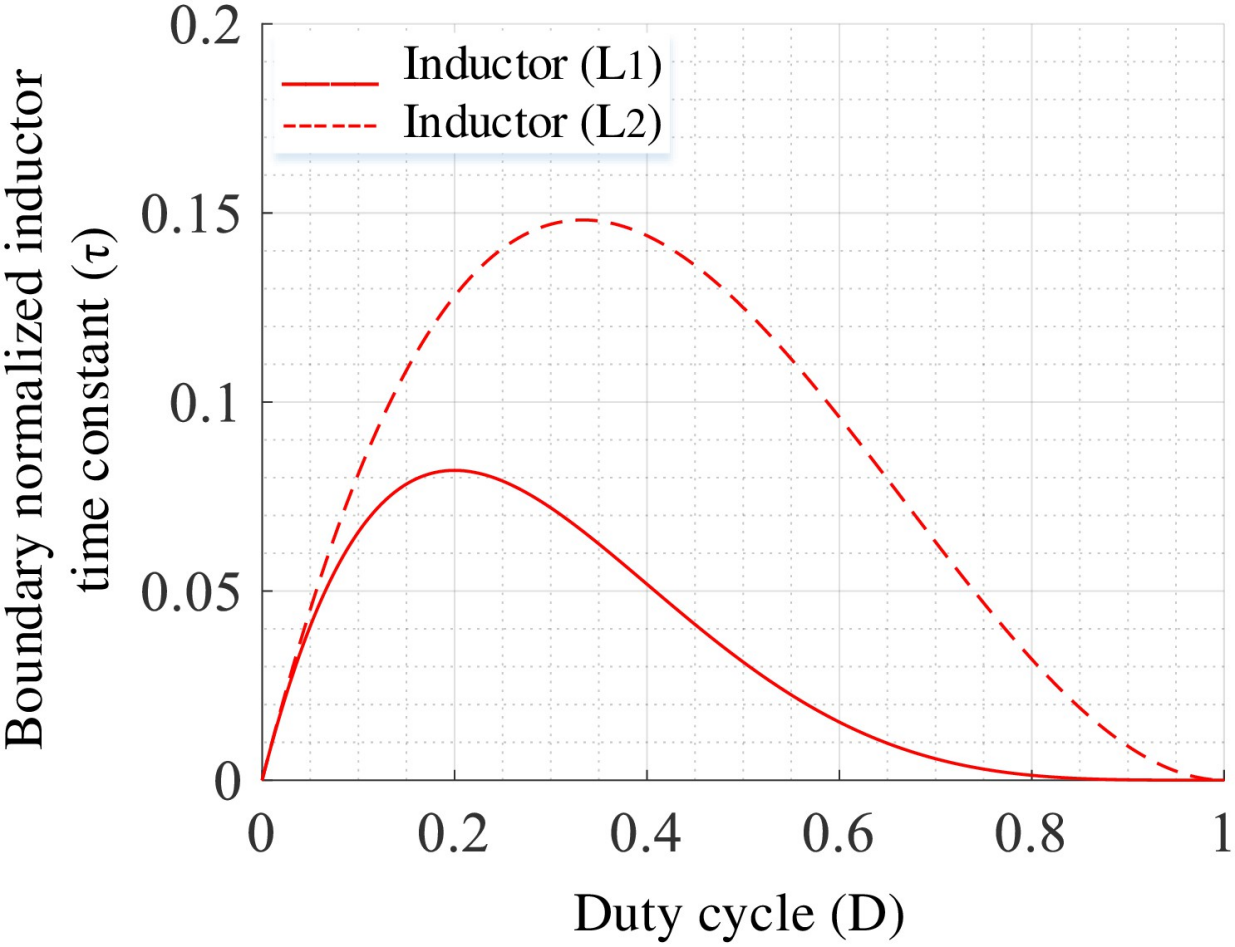
Boundary condition curves.

The operating mode of the converter varies based on the region as shown in Fig 7 and the continuous or discontinuous current mode (i.e., CCM or DCM) operations will be determined by the following conditions:

$$\begin{array}{c} \tau >\tau_{B}\;\;\text{CCM}\;\text{mode} \end{array}$$ (49)

$$\begin{array}{c} \tau <\tau_{B}\;\;\text{DCM}\;\text{mode} \end{array}$$ (50)

$$\begin{array}{c} \tau =\tau_{B}\;\;\text{Boundary}\;\text{mode} \end{array}$$ (51)

The following section presents comparisons of this new topology against topologies in the similar frame (i.e. high gain).

## 3 Comparisons with similar topologies

**Fig 8:**
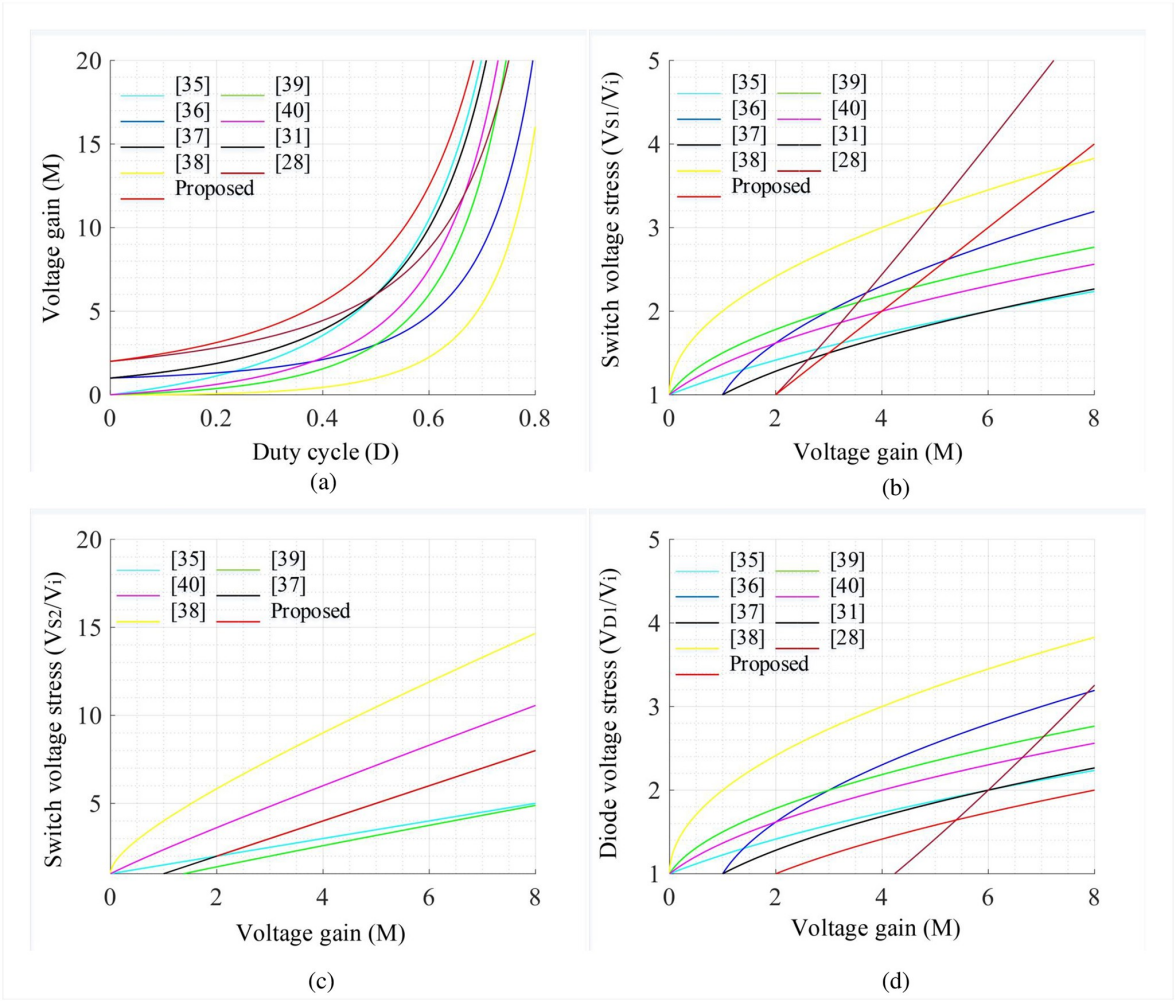
Comparative performance curves: (a) *D* vs *M*, (b) *D* vs *V*_*S*1_/*V*_*i*_, (c) *D* vs *V*_*S*2_/*V*_*i*_, and (d) *D* vs *V*_*D*1_/*V*_*i*_.

**Fig 9:**
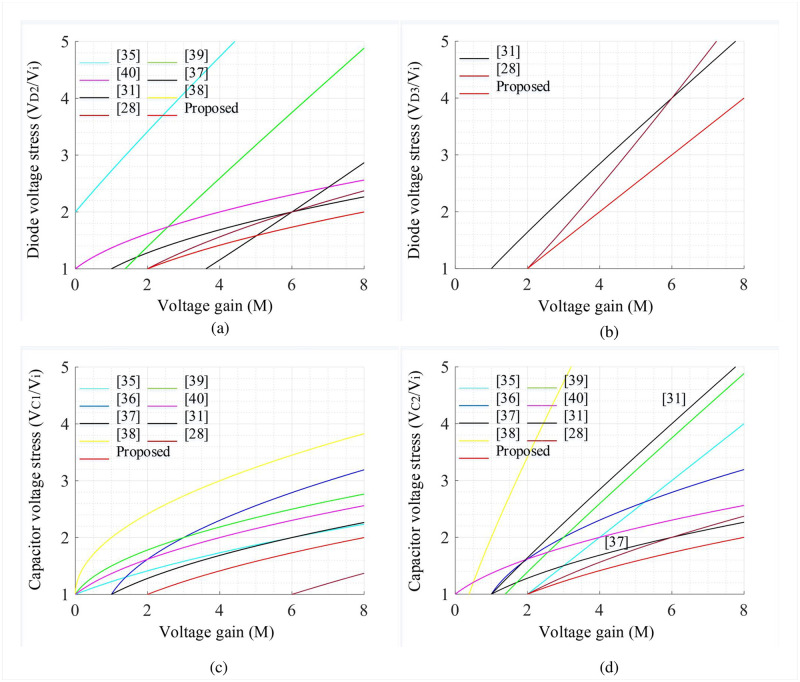
Comparative performance curves: (a) *D* vs *V*_*D*2_/*V*_*i*_, (b) *D* vs *V*_*D*3_/*V*_*i*_, (c) *D* vs *V*_*C*1_/*V*_*i*_, and (d) *D* vs *V*_*C*2_/*V*_*i*_.

The salient features of the proposed converter along with recently suggested traditional converters in a similar frame are summarized in Table 1. Moreover, Figs 8, 9 shows the comparative analysis in which the proposed converter is compared against conventional converters to attain a voltage conversion ratio of 8. While attaining the voltage gain of 8, different converters require different duty cycles to drive the converter. Consequently, the voltage stress of different components varies which defines the overall performance of the respective converter. In Fig 8(a), the voltage gain of topologies presented in [28, 31, 35–40] are compared with the proposed one where the proposed one is superior as compared to all conventional topologies. To achieve such a higher voltage gain, the converter studied in [35] utilized 14 components which is the highest count. The topologies in [31, 39] and the proposed converter utilized 12 components. However, for having a gain of 8, the converters [36, 38–40] used a duty cycle between 61% to 74% whereas, the converters [28, 31, 35, 37] need less duty cycle compared to [36, 38–40] which lies between 55% to 58%. In contrast, the proposed topology requires the lowest duty cycle, i.e., 50% compared to all conventional models. Hence, the voltage stress profiles of several components, e.g., semiconductor switches, diodes, and capacitors can be reduced significantly. The voltage stress comparisons of switches (*S*_1_ and *S*_2_) for different quadratic boost converters are depicted in Fig 8(b) and 8(c) which demonstrate that there is a moderate switch voltage stress for (*S*_1_) along with an improved outcome for (*S*_2_) compared to [28, 36, 38, 40], respectively. However, the converter suggested in [35] has the lowest voltage stress for both switches. On the other hand, the voltage stress on the diode (*D*_1_, *D*_2_, and *D*_3_) can be observed from Figs 8d and 9(a) and 9(b) in which the stress generated on all three diodes is less for the proposed converter compared to all the conventional converters. In addition, the stress voltage of capacitors (*C*_1_ and *C*_2_) is also less for the proposed converter while comparing to its counterparts according to the analysis depicted in Fig 9(c) and 9(d). The comparison of the remaining two features such as continuous source current and common ground facility can be checked from Table 1, which clearly states that the proposed converter exhibits both features whereas the only converter in [31] has no common ground feature on its structure. Another important facility is the same switch ground which eliminates the need for a separate DC power supply for the semiconductor gate driver circuit. The converters suggested in [35–40] utilized two semiconductor switches on the structure where the ground point of both the switches are not tied to the same point and hence, separate power supplies are required for individual switches. As a result, the overall system cost of conventional converters increases. However, the proposed converter has the same switch ground which ultimately omits the requirement of a separate power supply and offers better flexibility to relevant applications.

**Table 1:** Comparisons with different topologies for same voltage gain, *M* = 8.

Topology	Ref. [35]	Ref. [36]	Ref. [37]	Ref. [38]	Ref. [39]	Ref. [40]	Ref. [31]	Ref. [28]	Proposed
*S*	2	2	2	2	2	2	1	1	2
*D*	3	3	3	2	3	3	4	4	4
*C*	5	3	3	3	4	3	4	4	3
*L*	4	2	2	3	3	2	3	2	3
*Total*	14	10	10	10	12	10	12	11	12
CSC	✓	✓	✓	✓	✓	✓	✓	✓	✓
SG	✓	✓	✓	✓	✓	✓	×	✓	✓
SSG	×	×	×	×	×	×	×	×	✓
*D*	0.555	0.687	0.560	0.739	0.640	0.610	0.560	0.580	0.500
$\frac{V_{0}}{V_{i}}$	$\frac{2D(2-D)}{{\left(1-D\right)}^{2}}$ 8.00	$\frac{1-D+D^{2}}{{\left(1-D\right)}^{2}}$ 8.00	$\frac{\left(1+D\right)}{{\left(1-D\right)}^{2}}$ 8.00	$\frac{D^{2}}{{\left(1-D\right)}^{2}}$ 8.00	$\frac{D(1+D)}{{\left(1-D\right)}^{2}}$ 8.00	$\frac{2D}{{\left(1-D\right)}^{2}}$ 8.00	$\frac{\left(1+D\right)}{{\left(1-D\right)}^{2}}$ 8.00	$\frac{\left(2-D\right)}{{\left(1-D\right)}^{2}}$ 8.00	$\frac{2}{{\left(1-D\right)}^{2}}$ 8.00
$\frac{V_{S1}}{V_{i}}$	$\frac{1}{\left(1-D\right)}$ 2.25	$\frac{1}{\left(1-D\right)}$ 3.20	$\frac{1}{\left(1-D\right)}$ 2.28	$\frac{1}{\left(1-D\right)}$ 3.84	$\frac{1}{\left(1-D\right)}$ 2.78	$\frac{1}{\left(1-D\right)}$ 2.57	$\frac{1}{\left(1-D\right)}$ 2.28	$\frac{1}{{\left(1-D\right)}^{2}}$ 5.67	$\frac{1}{{\left(1-D\right)}^{2}}$ 4.00
$\frac{V_{S2}}{V_{i}}$	$\frac{1}{{\left(1-D\right)}^{2}}$ 4.94	$\frac{\left(3D-2\right)}{{\left(1-D\right)}^{2}}$ 0.63	$\frac{\left(1+D\right)}{{\left(1-D\right)}^{2}}$ 8.00	$\frac{1}{{\left(1-D\right)}^{2}}$ 14.7	$\frac{D}{{\left(1-D\right)}^{2}}$ 4.94	$\frac{\left(1+D\right)}{\left(1-D\right)}$ 10.59	×	×	$\frac{2}{{\left(1-D\right)}^{2}}$ 8.00
$\frac{V_{D1}}{V_{i}}$	$\frac{1}{\left(1-D\right)}$ 2.25	$\frac{1}{\left(1-D\right)}$ 3.20	$\frac{1}{\left(1-D\right)}$ 2.28	$\frac{1}{\left(1-D\right)}$ 3.84	$\frac{1}{\left(1-D\right)}$ 2.78	$\frac{1}{\left(1-D\right)}$ 2.57	$\frac{1}{\left(1-D\right)}$ 2.28	$\frac{D}{{\left(1-D\right)}^{2}}$ 3.29	$\frac{1}{\left(1-D\right)}$ 2.00
$\frac{V_{D2}}{V_{i}}$	$\frac{\left(2-D\right)}{{\left(1-D\right)}^{2}}$ 7.16	$\frac{\left(1-2D\right)}{\left(1-D\right)}$ 1.12	$\frac{1}{\left(1-D\right)}$ 2.28	$\frac{D}{{\left(1-D\right)}^{2}}$ 10.85	$\frac{D}{\left(1-D\right)}$ 4.94	$\frac{1}{\left(1-D\right)}$ 2.57	$\frac{1}{\left(1-D\right)}$ 2.34	$\frac{D}{{\left(1-D\right)}^{2}}$ 2.90	$\frac{1}{\left(1-D\right)}$ 2.38
$\frac{V_{D3}}{V_{i}}$	×	×	×	×	×	×	$\frac{1}{{\left(1-D\right)}^{2}}$ 5.17	$\frac{1}{{\left(1-D\right)}^{2}}$ 5.67	$\frac{1}{{\left(1-D\right)}^{2}}$ 4.00
$\frac{V_{C1}}{V_{i}}$	$\frac{1}{\left(1-D\right)}$ 2.25	$\frac{1}{\left(1-D\right)}$ 3.20	$\frac{1}{\left(1-D\right)}$ 2.28	$\frac{1}{\left(1-D\right)}$ 3.84	$\frac{1}{\left(1-D\right)}$ 2.78	$\frac{1}{\left(1-D\right)}$ 2.57	$\frac{1}{\left(1-D\right)}$ 2.28	$\frac{D}{\left(1-D\right)}$ 1.38	$\frac{1}{\left(1-D\right)}$ 2.00
$\frac{V_{C2}}{V_{i}}$	$\frac{D(2-D)}{{\left(1-D\right)}^{2}}$ 4.04	$\frac{1}{\left(1-D\right)}$ 3.20	$\frac{1}{\left(1-D\right)}$ 2.28	$\frac{D}{{\left(1-D\right)}^{2}}$ 10.85	$\frac{D}{{\left(1-D\right)}^{2}}$ 4.94	$\frac{1}{\left(1-D\right)}$ 2.57	$\frac{1}{{\left(1-D\right)}^{2}}$ 5.17	$\frac{1}{\left(1-D\right)}$ 2.38	$\frac{1}{\left(1-D\right)}$ 2.00

## 4 Power loss calculations

Power losses for the proposed topology are calculated using the equivalent model where all parasitic resistances are considered to represent the non-ideal conditions. The overall power loss within the proposed converter is due to the losses of inductors, capacitors, diodes, and semiconductor switches. The inductor current and capacitor voltage ripples are not counted for simplifying the analysis. The total switching loss can be divided into conduction and switching losses. If (*r*_*ds*_) represents resistance during the ON time, the conduction loss can be calculated using this whereas the switching loss is due to the ON and OFF states of switches. Here, the switching loss is neglected as it is significantly low. The conduction loss can be calculated by using Eqs (17)–(21) with (*r*_*S*1_) and (*r*_*S*2_) as resistances for *S*_1_ and *S*_2_, respectively when these switches are ON. Representing the corresponding power losses by *P*, the conduction losses for switches can be calculated as follows:

$$\begin{array}{c} I_{S1(rms)}=\frac{4I_{0}}{\sqrt{D}{\left(1-D\right)}^{2}} \end{array}$$ (52)

$$\begin{array}{c} P\left(r_{S1}\right)=I_{S1(rms)}^{2}r_{S1}=\frac{16}{D{\left(1-D\right)}^{4}}\frac{r_{S1}}{R}P_{0} \end{array}$$ (53)

$$\begin{array}{c} I_{S2(rms)}=\frac{I_{0}}{\sqrt{D}\left(1-D\right)} \end{array}$$ (54)

$$\begin{array}{c} P\left(r_{S2}\right)=I_{S2(rms)}^{2}r_{S2}=\frac{1}{D{\left(1-D\right)}^{2}}\frac{r_{S2}}{R}P_{0} \end{array}$$ (55)

Furthermore, the diode power loss can be written as follows:

$$\begin{array}{c} I_{D1(rms)}=\frac{2I_{0}}{\sqrt{D}{\left(1-D\right)}^{2}} \end{array}$$ (56)

$$\begin{array}{c} P_{rD1}=I_{D1(rms)}^{2}r_{D1}=\frac{4}{D{\left(1-D\right)}^{4}}\frac{r_{D1}}{R}P_{0} \end{array}$$ (57)

$$\begin{array}{c} P\left(v_{F1}\right)=\frac{2}{{\left(1-D\right)}^{2}}\frac{v_{F1}}{V_{0}}P_{0} \end{array}$$ (58)

$$\begin{array}{c} I_{D3(rms)}=\frac{2I_{0}}{\sqrt{D}D\left(1-D\right)} \end{array}$$ (59)

$$\begin{array}{c} P_{rD3}=I_{D3(rms)}^{2}r_{D3}=\frac{4}{D^{3}{\left(1-D\right)}^{2}}\frac{r_{D3}}{R}P_{0} \end{array}$$ (60)

$$\begin{array}{c} P\left(v_{F3}\right)=\frac{2}{D(1-D)}\frac{v_{F3}}{V_{0}}P_{0} \end{array}$$ (61)

The power losses due to inductors and capacitors can be computed as:

$$\begin{array}{c} P_{L}=I_{L(rms)}^{2}r_{L}\;\&\;P_{C}=I_{C(rms)}^{2}r_{C} \end{array}$$ (62)

With all these power losses, the total power loss can be determined as:

$$\begin{array}{c} P_{\left(Loss-Total\right)}=P_{S1}+P_{S2}+P_{D1}+P_{D2}+P_{D3}+P_{D0}+P_{L1}+P_{L2}+P_{L2}+P_{L3}+P_{C1}+P_{C2}+P_{C0} \end{array}$$ (63)

Finally, the expression for the proposed converter’s efficiency will be as:

$$\begin{array}{c} \eta =\frac{P_{0}}{P_{0}+P_{\left(Loss-Total\right)}}\times 100\% \end{array}$$ (64)

Based on all these, the simulation and experimental validations are carried out in the next section.

## 5 Simulation and experimental results

**Fig 10:**
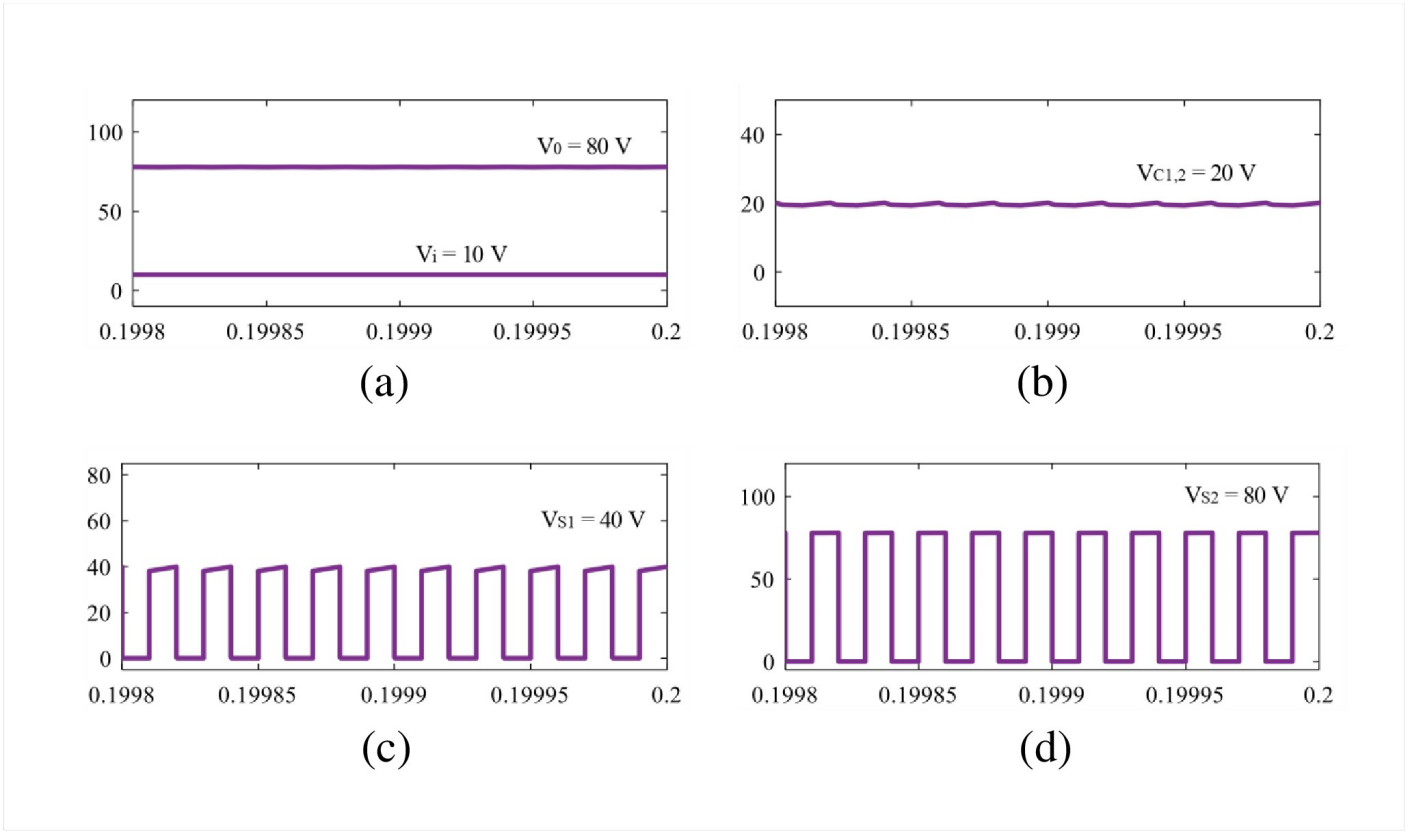
Simulation results: (a) *V*_*i*_, and *V*_0_,(b) *V*_*C*1,2_, (c) *V*_*S*1_ and (d) *V*_*S*2_.

**Fig 11:**
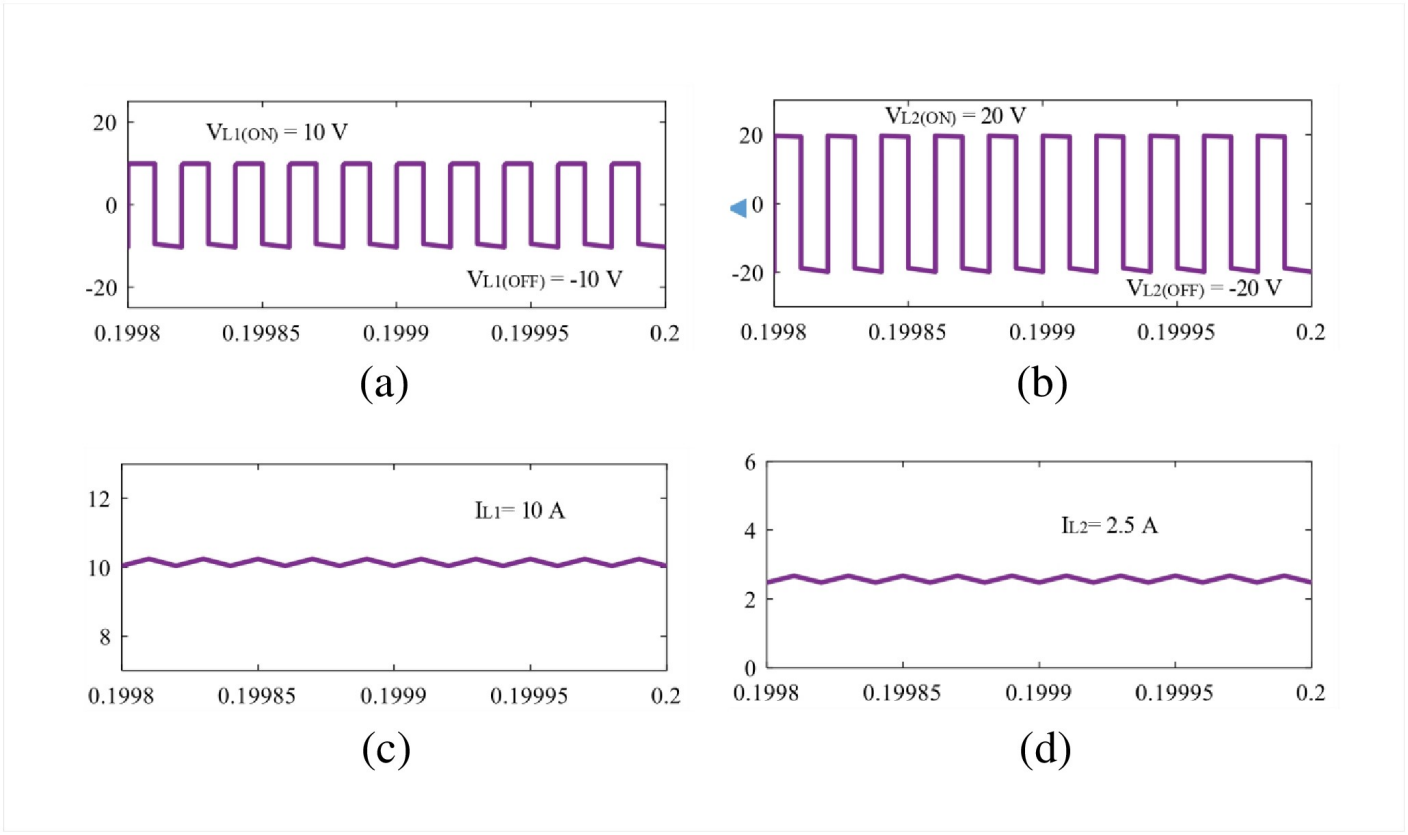
Simulation results: (a) *V*_*L*1_, (b) *V*_*L*2,3_, (c) *I*_*L*1_, and (d) *I*_*L*2,3_.

**Fig 12:**
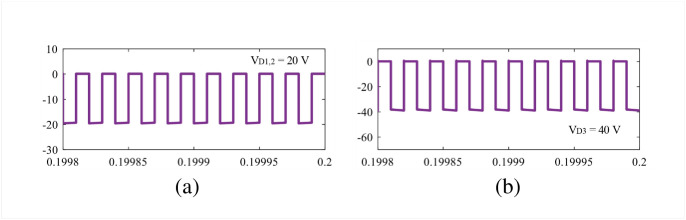
Simulation results: (a) *V*_*D*1,2_ and (b) *V*_*D*3_.

The theoretical claims are validated in this section using simulation and experimental results. The parameters are selected as: *L*_1_ = 1 mH, *L*_2,3_ = 490 *μ*H, C = 1000 *μ*F, and resistance, R = 64 Ω for both simulation and experiment. The gate signals are generated using the texas instrument DSP TMS320F28027F and gate driver IC TLP-250H. The simulation results are discussed in the next subsection based on Figs 10–12.

### 5.1 Simulation results

The simulation studies for the newly proposed structure are carried out in Matlab/Simulink environment. In the simulation, the input voltage is set as 10 V to achieve a voltage gain of 8 at 0.5 duty cycle, according to Eq (10). From Fig 10(a), it is obvious that the new structure reaches an output voltage of 80 V where the capacitors *C*_1_ and *C*_2_ experience the same voltage, *V*_*C*1_ = *V*_*C*2_ = 20 V as shown in Fig 10(b). During the time, the capacitor voltage stress of the proposed converter becomes 2 according to Eq (9). The simulation results align with the theoretical findings illustrated in Eqs (10) and (9). In Fig 10(c) and 10(d), the voltage across semiconductor switches *S*_1_ and *S*_2_ are depicted, from where it can be noticed that the switch *S*_1_ experiences voltage of 40 V, i.e., *V*_*S*1_ = 40 V. Furthermore, the switch voltage *V*_*S*2_ is found as the same of the output voltage, i.e., 80 V. On the other hand, the inductor voltage during the charging and discharging period and average current can be found in Fig 11(a)–11(d). The voltages *V*_*L*1(*ON*)_ = 10 V and *V*_*L*1(*OFF*)_ = −10 V for inductor *L*_1_ and *V*_*L*2(*ON*)_ = 20 V and *V*_*L*2(*OFF*)_ = −20 V for inductor *L*_2_ are shown in Fig 11(a) and 11(b). The voltages are also same for the inductor *L*_3_ as per the simulation and theoretical modeling. From the Fig 11(c) and 11(d), the average currents are found as 10 A and 2.5 A for inductors *L*_1_ and *L*_2,3_, respectively. At the same time, the diode *D*_1_ becomes reverse-biased during the ON state for which *V*_*D*1_ = 20 V. In contrast, the diodes *D*_2_, *D*_3_ become reverse biased during the OFF time, and the diode voltages are found *V*_*D*2_ = 20 V and *V*_*D*3_ = 40 V, respectively. All simulation results shown in Figs 10 to 12 match well with the theoretical relationship expressed in Eqs (12)–(16).

### 5.2 Experimental results

**Fig 13:**
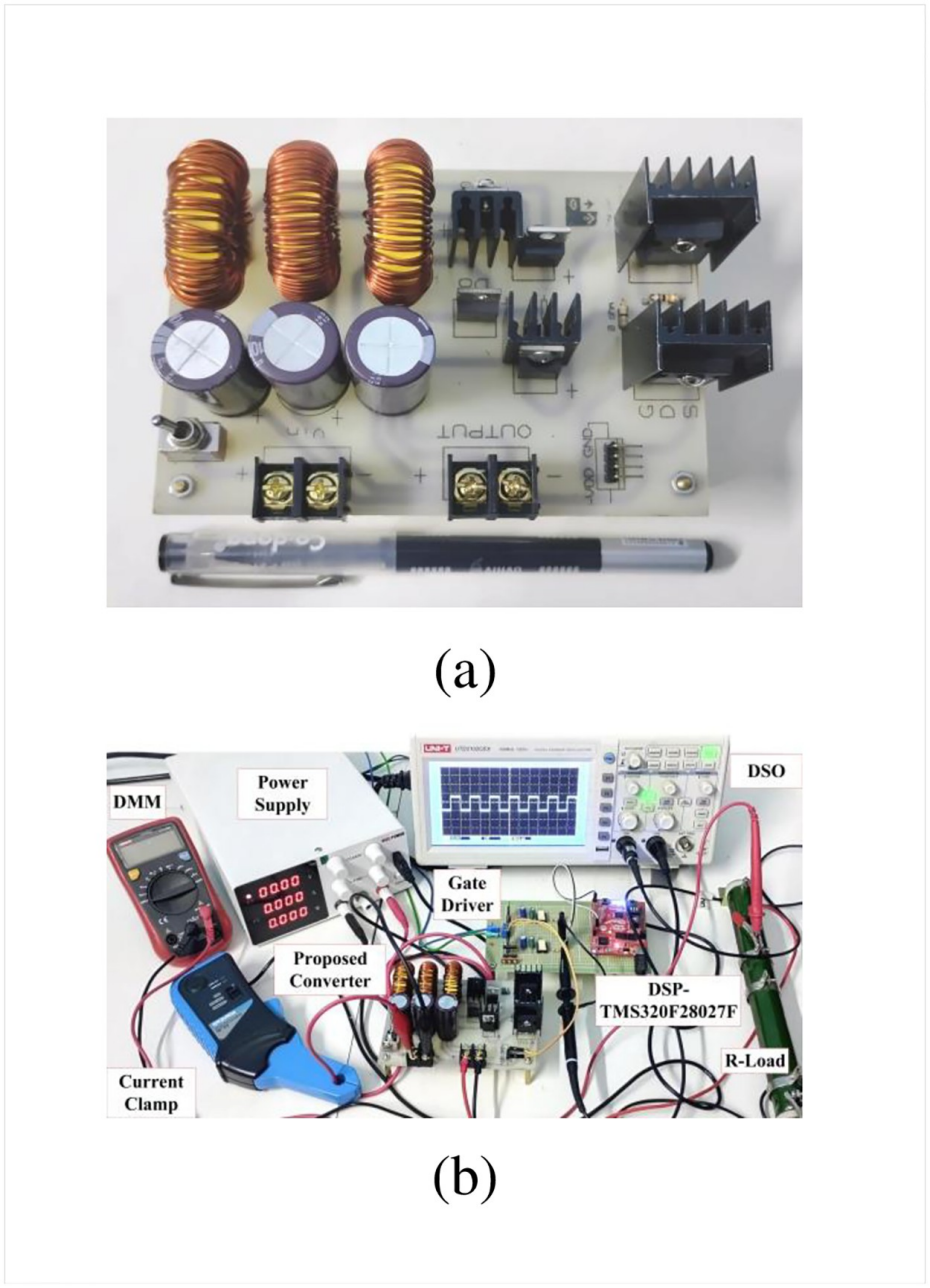
(a) Prototype and (b) Experimental setup.

**Fig 14:**
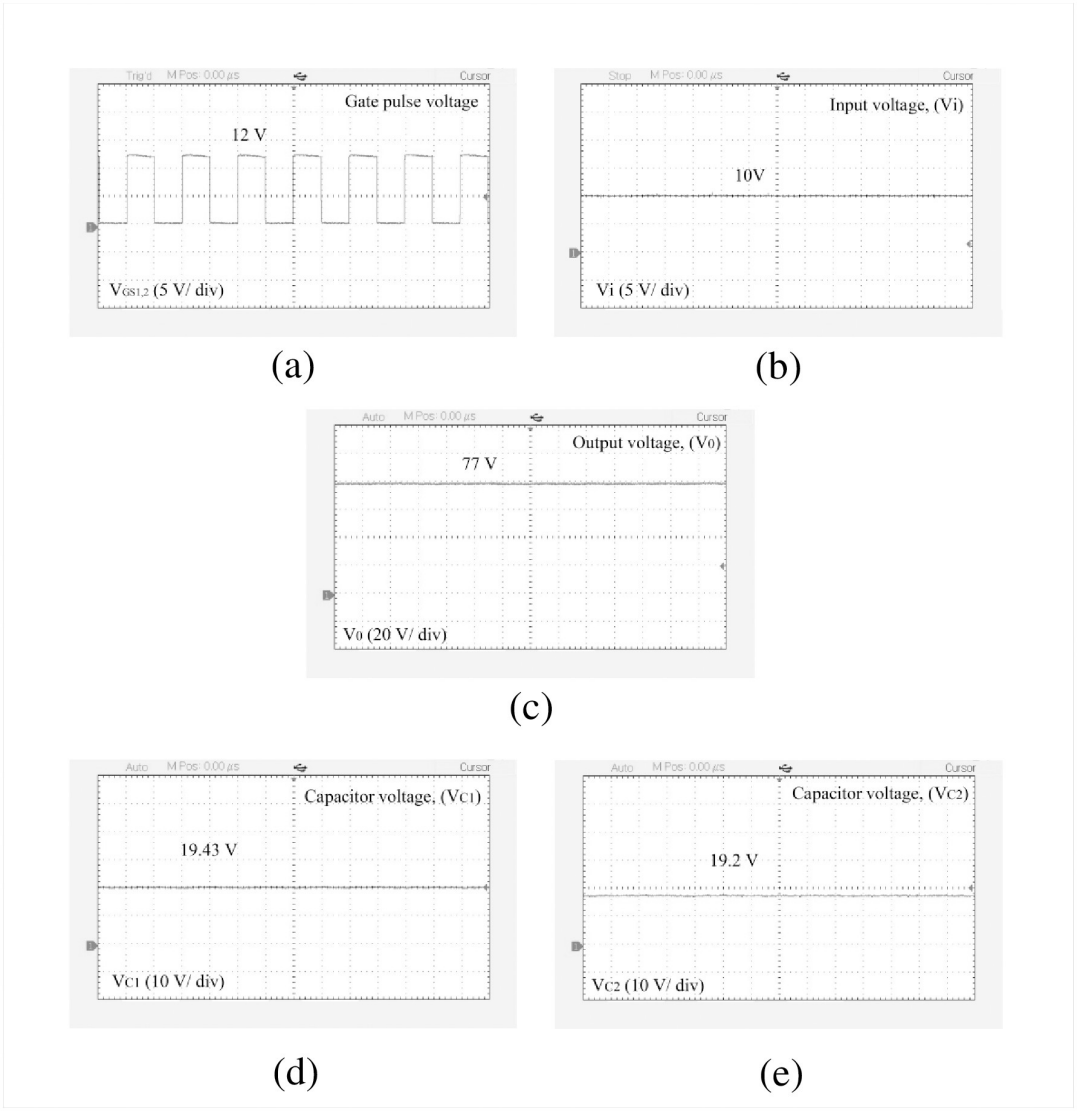
Experimental results: (a) Gate pulse, (b) Voltage at the input, (c) Voltage at the output, (d) Voltage across capacitor (*C*_1_), and (e) Voltage across capacitor (*C*_2_).

**Fig 15:**
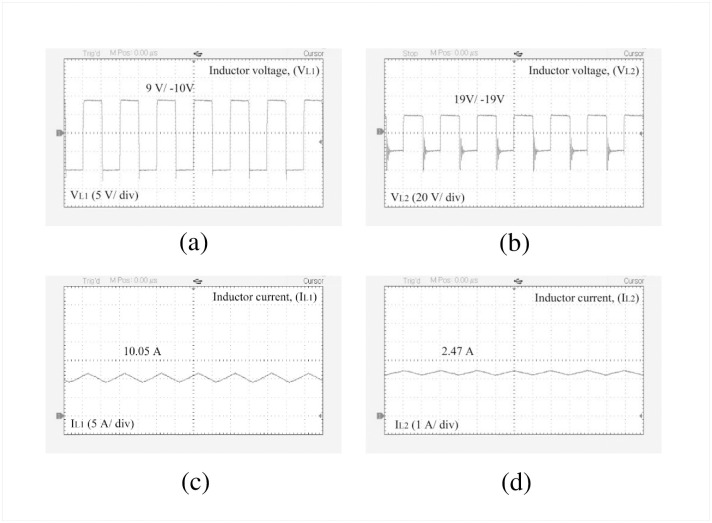
Experimental results: (a) Voltage across *L*_1_, (b) Voltage across *L*_2_, (c) Current through *L*_1_, and (d) Current through *L*_2_.

**Fig 16:**
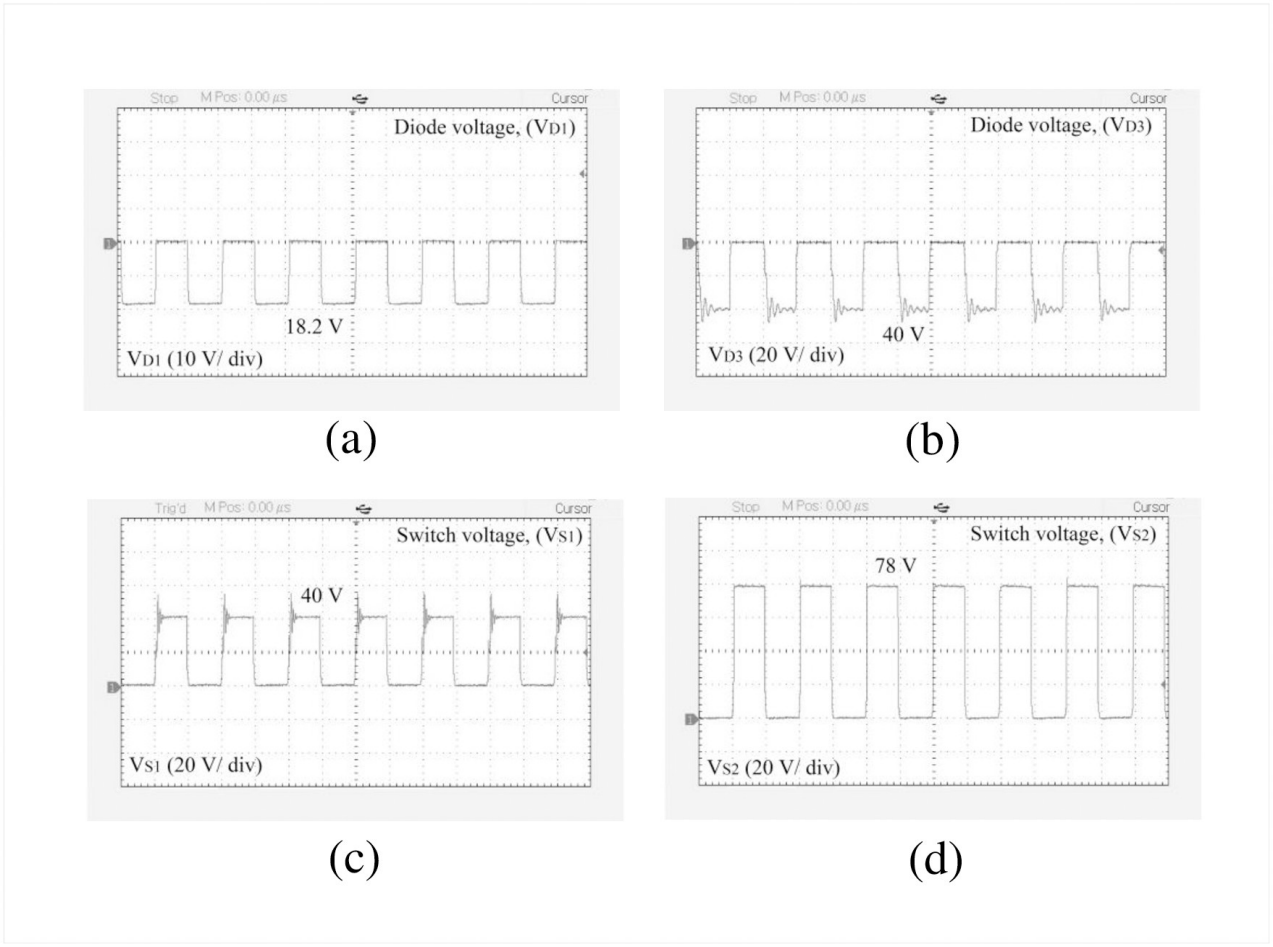
Experimental results: (a) Diode (*D*_1_) voltage, (b) Diode (*D*_3_) voltage, (c) Switch (*S*_1_) voltage, and (d) Switch (*S*_2_) voltage.

**Fig 17:**
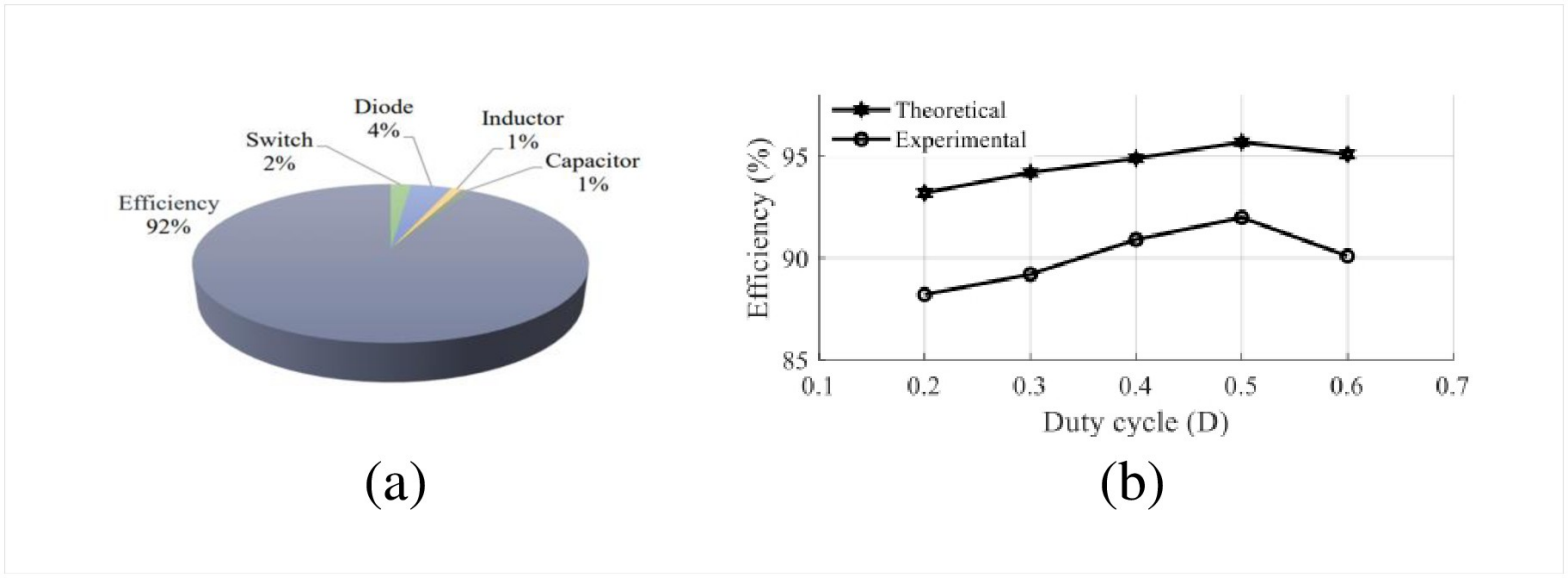
Comparative performance curves: (a) Power loss distribution, (b) Efficiency curves.

The performance of the designed high-gain quadratic topology is experimentally validated using a 250 W laboratory prototype as shown in Fig 13(a) and experimental setup has been depicted in the Fig 13(b). The PWM signals for both active switches are generated using a TMS320F28027F DSP controller. To drive the gate signal of the semiconductor switch, the TLP250H-based optocoupler circuit is used which also serves the isolation purpose for the power and control circuits. In Fig 14(a), the PWM pulse is shown for a duty ratio of 0.5. Fig 14(b) to 14(e) shows the input voltage, output voltage, and capacitor voltages (i.e., for *C*_1_ and *C*_2_), respectively. When the input voltage applied to the proposed structure is 10 V, the output voltage reached nearly 77 V according to Fig 14(c). At the same time, the capacitor voltage across *C*_1_ and *C*_2_ are found 19.43 V and 19.2 V respectively as shown in Fig 14(d) and 14(e). From Fig 14(c), it is evident that the output voltage of the proposed converter maintains a close relationship with the theoretical and simulated values as per Eq (10) and Fig 10. Besides, voltages across *L*_1_ and *L*_2_ can be found from Fig 15(a) and 15(b) where *V*_*L*1_ is 9 V and −10 V during the charging and discharging operations. Similarly, inductor voltage for *L*_2_ was found around 20 V and −20 V, respectively. The results were observed same for the voltage across the inductor (*L*_3_). During this time, the inductor *L*_1_ draws a current of around 10.02 A which can be seen from Fig 15(c). The current for the inductor *L*_2_ was roughly around 2.47 A as depicted in Fig 15(d). The diode voltages for *D*_1_, and *D*_3_ can be observed in Fig 16(a) and 16(b) where *V*_*D*1_ = 18.2 V, and *V*_*D*3_ = 40 V respectively. A similar voltage like *V*_*D*1_ across diode *D*_2_ was observed in the experimental outcome. There is a slight drop in the diode voltage due to the parasitic effect of the device. In addition, the semiconductor switch voltages for *S*_1_ and *S*_2_ are shown in Fig 16(c) and 16(d) where *V*_*S*1_ = 40 V and *V*_*S*2_ = 78 V, respectively. All experimental results nearly align with the theoretical as well as simulation outcomes. However, a few discrepancies were encountered in the experimental results because of the non-ideal behaviors of the devices. The output voltage of the converter has a slight voltage drop of around 3.75% compared to the simulation outcome where the parameters taken were ideal. Please take note that due to the associated diodes and stitches being practical devices in the structure, the output voltage dropped slightly. The experiments are conducted to extract the practical voltage gain along with the efficiency. Considering a non-ideal condition of the designed topology and using Eqs (52)–(55) to (64), the power loss analysis is conducted which shows that diodes contributed to the highest percentage of the overall power loss that is around 4% and the semiconductor switches come after that for which it is nearly 2%. Both the inductor and capacitors hold an equal amount of power loss, i.e., around 1% of the total loss according to Fig 17(a). The theoretical and experimental efficiency of the designed topology reaches its highest value of 92% at 0.5 duty cycle as shown in Fig 17(b). Moreover, the efficiency is low at a lower duty ratio and starts to decrease again after 0.5. According to the overall result analysis and outcome, the newly designed converter offers promising efficiency during the operation.

## 6 Conclusion

An enhanced quadratic DC-DC converter topology having a non-isolated and high gain feature is designed which also has a less number of components on its structure. The voltage stress on the capacitor, diode, and switch are comparatively lower which ensures that components with lower rated voltage can be utilized to make the converter cost-effective while reducing the weight and size. Although the voltage stress of switch *S*_2_ is more compared to *S*_1_ but provides competitive performance compared to the conventional topologies. Furthermore, the designed topology draws continuous input current which omits the requirement of having an input filter in the system. Besides, the proposed converter has successfully omitted the separate control supply ground requirement by ensuring the same switch ground on its structure. Based on analyses, the proposed topology offered a maximum efficiency of 92% at 0.5 duty cycle with a 250 W experimental setup. All findings from different perspectives (i.e., theory, simulation, and experiment) are found consistent and thus, the proposed converter demonstrated its suitability for applications that require high-gain DC-DC boost converters.

## Supporting information

S1 File (7Z)
